# Why We Should Recognize AI as an Inventor

**DOI:** 10.1007/s11673-025-10429-4

**Published:** 2025-07-21

**Authors:** A. S. Bayındır, J. Danaher

**Affiliations:** University of Galway, University Road, Galway, H91 TK33 Ireland

**Keywords:** Artificial intelligence, AI, Research and development, R&D, Inventorship

## Abstract

It is now possible for AI systems to generate novel inventions without meaningful human direction and control. Should such inventions be patented? The prevailing consensus, confirmed in recent test cases and official guidance, is that patent law only covers inventions by natural persons (i.e., humans). This, however, sometimes creates an odd situation in which AI-generated inventions cannot be patented, nor can the humans responsible for those systems gain patent rights indirectly through the operation of the law. In this article, we argue against this prevailing consensus. We present five reasons for thinking that AI-generated inventions should be patentable and that AI systems should be legally recognized as inventors. In making this argument, we do not claim that modern AI systems have acquired some significant legal or moral status that is equivalent to humans. Our argument is more practical in nature. We argue that failing to recognize AI inventorship will have negative repercussions for economic development and innovation, at a time when AI assistance is needed.

## Introduction: DABUS and the Legal Debate About Inventorship

Innovation is crucial to modern economic and social life. Patents foster and maintain innovation by granting monopolistic rights, for a certain period of time, to those that invent technical solutions for particular problems (Fisher 2007). This grant of monopolistic rights is made in exchange for making the invention public and understandable to an average person skilled in the relevant art (Nard 2008; Waite 1920).

In most legal systems, the original inventors are the ones entitled to apply for and be the owners of a patent. However, other entities, who are not the primary inventors, are sometimes granted rights to apply for a patent by the operation of law. Employers, for example, are often given the right to apply for patents for the inventions that their employees have created under the terms of their employment contracts. But even though employers have the right to apply for a patent in such circumstances, according to the laws that are in force today, it is obligatory to write the identity information of the original inventor in a patent application (Fraser 2016).

This is because the correct determination of the identity of the inventor is crucial in allocating the moral rights arising from a patent and in determining who owns the right to apply for a patent. An employer, for instance, can only apply for a patent on behalf of their employee if a non-employee was the original inventor, the employer would not have that right.

This system has operated reasonably well in a world of human-only inventorship. However, recent developments in AI, in particular their incorporation into R&D processes, and their potential to generate inventions of their own, calls into question the validity of this approach and prompts a reconsideration of the existing legal position.

The various test cases brought by Stephen Thaler on behalf of his AI system—DABUS—are an illustration of this. Thaler is a computer scientist. He created the DABUS AI system to assist in the process of invention. He claims that DABUS, unaided and undirected by him, has generated at least two patent-worthy inventions—a food container based on fractal geometry and a type of flashing emergency beacon. He has applied for patents for both in various jurisdictions around the world. In all cases, he has asked for DABUS to be legally recognized as the original inventor and for himself to own the resulting patent by operation of law.

To date, with the sole exception of South Africa,^1^ none of the authorities in charge of the patent system have accepted his claims. For example, consider the decision of the U.K. Supreme Court. In December 2023, the court held that the *Patents Act 1977* could only be interpreted as recognizing natural persons as potential inventors. Since Thaler did not himself claim to be the original inventor, this meant that DABUS’s two inventions could not be patented under English law.^2^ This meant that Thaler could not own any patent rights under operation of law. This resulted in the odd position in which two items were invented, but neither of them could be legally protected as such.

Thaler’s applications to the European Patent Office and the United States Patent and Trademark Office (USPTO) were rejected on similar grounds. The Boards of Appeal of The European Patent Office held that an inventor can only be a natural person within the meaning of the European Patent Convention, and the United States Court of Appeals for the Federal Circuit held an inventor can only be an individual within the meaning of the Patent Act (35 U.S. Code) and that an individual means a natural person within the meaning of U.S. case law.^3^ The United State Supreme Court then denied a petition for a writ of certiorari in this case, thereby allowing the lower court’s decision to prevail.^4^

Following this, in February 2024, the USPTO confirmed the prevailing consensus when they issued inventorship guidance for AI-assisted inventions. The guidance concluded that while AI-assisted inventions are not prima facie unpatentable, only humans could be recognized as inventors, for legal purposes, because patents incentivize and reward human ingenuity (USPTO 2024).

In this article, we argue against this prevailing consensus and claim that AI-generated inventions can and should be protected under patent law and that AI systems should be recognized, where appropriate, as the original inventors in the resulting patent applications. This would mean that humans or human-owned enterprises would continue to be the patent owners, through operation of the law, but AI systems would be the original legally recognized inventors, appropriately identified as such in the associated patent documents.

## Why We Should Recognize AI as an Inventor

In making this argument, we do not presuppose some controversial view about the ontological or moral status of contemporary AI. We are not, for instance, claiming that AI has the same properties as natural persons, nor that they should be granted equivalent legal and moral standing. Our argument is more practical and economic in nature. All we assume is that (at least some) contemporary AI systems are capable of generating designs and technical specifications without the immediate direction and control of humans. This is not a controversial assumption. It is widely accepted that modern AI systems can learn, adapt, and generate outputs that are both surprising to and unforeseeable by their original creators (Abbott 2016; Pearlman 2018; Alpaydın 2020; Kissinger, Schmidt, and Huttenlocher 2021). This is, indeed, the property of modern AI systems that has generated such a rich literature on responsibility gaps arising from their use (Matthias 2004; Santoni de Sio and Mecacci 2021). It is also what motivates Thaler in his test cases and what is implied by the USPTO guidance suggesting that AI systems could generate inventions without meaningful human control and direction (USPTO 2024).

The AI system called AlphaFold, developed by Google DeepMind (Senior et. al 2020) is a good illustration of this. In 2018, it found a three-dimensional target protein in a drug development task and outperformed ninety-eight other competitors (Abbott 2020). Subsequently, AlphaFold solved a fifty-year-old grand challenge in biology in 2020, and its inventors were subsequently awarded the Nobel Prize in Chemistry 2024 in recognition of this discovery. But they didn’t make the discovery themselves; they created the AI system that made the discovery.

Based on this evidence, AI has the potential to transform research and development more profoundly if it becomes the standard practice for R&D processes. This, we suggest, could be best enabled by patenting AI-generated inventions and recognizing AI systems themselves as the original inventors. We present five main arguments for this: (i) patent laws should focus on the final product rather than the invention generation process; (ii) failing to recognize and patent AI-generated inventions is inconsistent with the innovation-promoting objectives of patent law; (iii) not patenting AI-generated inventions might lead parties to conceal AI systems’ intervention in the invention generation process, which could have a number of negative implications; (iv) rejecting AI inventorship may discourage the public disclosure of AI-generated inventions, which is one of the chief virtues of the patent system; (v) rejecting AI inventorship will discourage the use of AI in the innovation process at a time when such usage is desirable.

We will now elaborate on each of these arguments.

### (i) Patent laws should not focus on the invention generation process but the final product

Patent laws deal with inventions and award them with patents because they are the final products that advance science, technology, and innovation (Nard 2008; Abbott 2020). Patent laws do not, and should not, take the invention-generation process into account when determining patentability. For example, whether the inventor found the technical solution on her/his own, on a long walk along a beach, in a dream, or in a laboratory setting with a group of people, does not matter from a legal perspective (Abbott 2020). The consequences for the resulting invention will be the same under the applicable patent laws.

This conclusion is supported by considering that the patentability criteria in most jurisdictions focus on the output and not the process of discovery. In most jurisdictions, there are three main criteria for patentability. They are *novelty*, having an *inventive step* (being *non-obvious* under U.S. law), and *industrial applicabilit*y (or *utility* under US law). Each of these criteria is focused on the nature of the invention itself, not the process leading up to it.

Refusing to patent AI-generated inventions, simply because they are created by a machine rather than a human, surreptitiously adds another criterion of human inventorship to the patentability criteria, which is not consistent with the goals of modern patent law. Modern patent law has moved away from any direct consideration of the processes underlying invention because it is recognized that this can lead to the arbitrary exclusion of some inventions from patent protection. As Abbot points out, in the 1950 s in the United States, a doctrine called “flash of genius” was used to determine the patentability of an invention (Abbott 2020). According to this doctrine, for an invention to be patented, it had to be developed as a result of a moment of inspiration by a natural person and not as a result of methodical laboratory research. As the subjective nature of this test was brought to the attention of lawmakers, this doctrine was abandoned. Subsequently, it was regulated that the method of conceiving an invention should not affect its patentability. An explicit provision to this effect was added to the Patent Act (35 U.S. Code) under Section 103 with the following text “Patentability shall not be negated by the manner in which the invention was made” (Abbott 2020).

This historical episode shows how patent law evolved in a way that focuses on inventions themselves, not on extraneous factors. This position should not be reversed by insisting on a human origin.

### (ii) Not patenting AI-generated inventions does not comply with the general purpose of patent laws which is fostering innovation

In the majority of the DABUS test cases discussed in the introduction, patent offices and courts have preferred a textual interpretation of patent law statutes in which the inventorship status of AI is rejected on the basis that only natural persons can be inventors. As noted, this creates the odd result whereby, if there is not a natural person that can claim inventorship status, the resulting inventions are unpatentable. This interpretation undermines the purpose of patent law. The purpose of the patent law is not and should not be promoting human-generated invention, but rather it is, and it should be, promoting innovation *for the benefit of humanity*. The grant of monopolistic rights is the carrot that is provided as the incentive for such innovation. If AI generated inventions cannot be patented, there is little incentive to create them or the underlying AI systems that enable them. Society loses out as a result (Abbott 2020; Tripathi and Ghatak 2017).

As a counterargument to this, it could be noted that, in their recent guidance, the USPTO states that the patent system is designed to encourage human ingenuity. They do this by referring to legal texts that were drafted at a time when the only source of ingenuity was human beings (USPTO 2024). We submit that those legal documents referred to human ingenuity not because only human ingenuity was useful for humanity, but because humans were the only source of ingenuity. We now live in a different world where different sources of ingenuity—in particular AI-generated ingenuity—exist. We should recognize and protect this new source too, if we care about incentivising innovation for the benefit of humanity.

### (iii) Not patenting AI-generated inventions might lead parties to hide the use of AI in the invention generation process

The preceding argument presumes that humans remain honest about their role in invention. But if denying patentability to AI-generated inventions becomes the norm, AI’s intervention in R&D processes might be concealed by the people who develop an innovative AI, or who have access to these systems. They might, in other words, pretend that they or some other humans were the original inventors in order to ensure that the resulting inventions are patentable. Such subterfuge may, already, be happening. The computer scientist and genetic programming pioneer John Koza, for instance, has suggested that he and his team have been awarded patents for inventions that were actually created by an AI by writing themselves as the inventors in the patent applications (Pearlman 2018; Abbott 2016; Schuster 2018).

Koza is to be commended for his honesty but if an AI’s role in the invention process is concealed, it will create an information asymmetry in the legal regulation of innovation. This asymmetry might eventually a) prevent patent authorities or legislatures from recognizing the need to update patentability criteria, which might in turn interfere with the efficient application of the patent system, rendering it ineffective in the promotion of innovation; b) create problems in the allocation of the moral rights arising from a patent; c) prevent patent authorities or legislatures from recognizing the need to update the duration of patent protection in light of the speed of AI technologies; and d) put an unnecessary pressure of cancellation on the patent owners who concealed AI’s intervention to the R&D processes. Let’s consider each of these problems in more detail.

First, some scholars argue that patentability criteria of AI-generated inventions should be different from the current patentability criteria (Kavuşturan 2020; Mammen and Richey 2020; Firth-Butterfield et al. 2018; Cubert and Bone 2018; Fraser 2016; Ali 2020; Samore 2013). In particular, they have argued that updating the “inventive step” or “non-obviousness” criterion is important because this criterion is typically assessed by reference to the “person having an ordinary skill in art” (de Rassenfosse, Jaffe, and Wasserman 2023; Shemtov and Gabison 2022). In other words, it is a human equivalency standard. This reference may not be useful in an era of AI-assisted innovation (de Rassenfosse, Jaffe, and Wasserman 2023; Shemtov and Gabison 2022). This is because the legal fiction of the “person having an ordinary skill in art” takes human beings and their capabilities as the standard when assessing whether the invention involves an inventive step or is non-obvious. If this criterion is directly applied to AI-generated inventions, the threshold for patentability might be too low, causing inefficiencies in the application of the patent system. This is because humans and AI systems possess different strengths and weaknesses. A conclusion that is non-obvious to a human might be obvious to an AI system, considering its ability to evaluate more possibilities in less time.

If AI-generated inventions are evaluated with the human non-obviousness standard in mind, then over-patenting (excessive patenting) may arise (Mandel 2008; Abbott 2020; Mazzi 2020). This could create barriers to entry to markets and hinder innovation (Firth-Butterfield et al. 2018; Fraser 2016). Granting patents creates a social and economic cost because of the monopolistic rights they entail. Legal systems bear this cost because of the social and economic benefit that the patent will provide by enabling the public disclosure of inventions (Fisher 2007; Mazzi 2020; Kavuşturan 2020; Gervais 2019; Samore 2013). For this reason, the threshold for granting patents should be adjusted by considering the features of AI systems in such a way that the inventive step criterion is neither too low, hindering innovation, nor too high, making it impossible to obtain patent protection for an invention (Mandel 2008; Abbott 2020; Fraser 2016; Ramalho 2017; Firth-Butterfield et al. 2018; Eisenberg 1999). In other words, the aim of the patent system should not just be solely granting patents for all inventions, but rather to grant patents for socially valuable inventions. If an invention can easily be generated by an AI system, society should not bear the cost of granting that patent. For this reason, a strong case can be made that the inventive step criterion should already be updated to factor in the realities of AI-assisted innovation. But if AI-inventorship is not honestly disclosed in patent applications, it will not be possible to effectively examine the patentability of the invention in question. If there is concealment, an invention that should not be entitled to patent protection may end up benefiting from unnecessary patent protection.

Second, if an AI’s use in the R&D processes is concealed, there will be problems with the allocation of the moral rights arising from inventorship. “Moral rights” are a term of art in intellectual property law. They refer to non-economic rights enjoyed by an author or inventor, specifically rights pertaining to the integrity of their work and their reputation (Lee 2023). Granting moral rights to a human who played no role in the specific invention is unnecessary and unjustified, but this is what will happen if humans are encouraged to conceal the use of AI. Additionally, this unfair allocation of moral rights has the potential to diminish the efforts of individuals who create inventions through processes not involving AI. Suspicion may arise that no one truly deserves the relevant moral rights if concealment is widely practiced. Human and AI-assisted innovation suffers as a result.

Third, concealing AI inventorship could result in a failure to appreciate the need to update the duration of patent protection in light of the speed of AI technologies. The general rule for patent protection is to grant patents for a period of twenty years. However, considering the pace of today’s technology, this patent period may not adequately balance the interests of patent owners and the public. Although there is a benefit that the public will enjoy from patent-protected inventions, the twenty-year period may be quite long given the rapid pace of today’s world. One of the advantages of using AI systems is that they could enable more efficient research in less time. Therefore, to establish a more equitable balance of benefits, the duration of patent protection might need to be shortened (Kavuşturan 2020; Fraser 2016). But we can only motivate this legal change if we have a clear and honest conception of the role of AI in the invention process. Concealing the role of AI prevents us from highlighting any imbalance in the current legal position.

Fourth, if people who are not originally inventors list themselves as inventors, they render themselves hostages to fortune. Patent owners are always under the pressure of a cancellation. This is because, in many legal systems today, various mechanisms are available for third parties to challenge the validity of a patent. One of the main ways they can do this is if they can prove that the named inventor was not the first to create the associated invention or that the invention was actually created by someone else (World Intellectual Property Organisation n.d.). But if this can happen when there are disputes over human inventorship, it can also happen when there are disputes over the exact role of an AI in the invention process. If a third party can prove that an AI and not a human was the true inventor, this could result in patents being revoked (Pearlman 2018). This would lead to further inefficiencies in the patent system, creating uncertainty about the validity and sustainability of a patent granted under such conditions. Given that patent law is already subject to much high-cost, and arguably nuisance, litigation (e.g., through the activity of so-called “patent trolls”) this is not a risk to be taken lightly.

These points seem particularly compelling in light of recent public recognition of the importance of AI in scientific discovery. In 2024, several of the laureates of the Nobel Prizes in Physics and Chemistry contributed either to the development of AI technologies or to their deployment in solving long-standing scientific problems, some of which have been worked on for over fifty years.

The Nobel Prize in Physics was awarded to John J. Hopfield and Geoffrey E. Hinton for their foundational discoveries and inventions that enabled machine learning with artificial neural networks. Meanwhile, as already noted, the Nobel Prize in Chemistry was awarded to David Baker for his work in computational protein design, alongside Demis Hassabis and John Jumper, who successfully utilized artificial intelligence to predict the structure of almost all known proteins.

These prizes trigger the questions of whether Hopfield and Hinton would have been granted this credit in physics if the applications of these machine learning technologies in chemistry had not been made obvious by Baker, Hassabis, and Jumper, or whether Baker, Hassabis, and Jumper could have been awarded if they had not disclosed that they deployed AI technologies in their work.

As the creation of new proteins and the prediction of protein structures are remarkable achievements, it could be argued that Baker, Hopfield, and Hinton might have won these awards even without disclosing their use of AI. However, this disclosure provided essential transparency and created a reference point for future discoveries and studies. If the role of AI in the discovery process had been hidden, science would suffer, and future discovery would be hindered.

Our argument is that recognizing AI technologies as inventors offers comparable benefits. Individual inventions are crucial, and patent protection makes these inventions public, preventing cost duplication and establishing a reference point for future R&D (Nard 2008; Fisher 2007; Fraser 2016; Kavuşturan 2020). These inventions contribute technical solutions to specific technical problems, which is critical for fostering innovation. However, with the development of AI technologies and their incorporation into R&D, inventorship itself has become another element requiring public disclosure. It is not about giving credit to the AI qua inventor, but to prevent cost duplication and to provide a reference for future research.

One obvious benefit to this is that, once competitors know about the use of the AI inventor, they may wish to employ these same systems in their companies. Transparency in inventorship will enable third parties to pursue licensing agreements allowing them to do so. This will likely increase the application of these technologies, enhancing the potential for generating more useful technical solutions.

It is true that assigning patent application rights will become more complex with the inclusion of AI technologies in these procedures. However, it is important to emphasize that granting inventorship to AI systems does not create this issue; rather, it clarifies and makes the complexities apparent, facilitating further actions to address the challenges introduced by their inclusion in research and development.

### (iv) Rejecting AI inventorship may discourage the public disclosure of AI-generated inventions

One of the objectives of patent law is to make inventions public (Nard 2008; Mazzi 2020). Publicity comes with the fear of expropriation. This fear is mitigated by the grant of monopolistic rights over an invention for a certain period of time. Public disclosure has significant benefits. Disclosure prevents inefficient doubling of the R&D costs for society, provides a reference point for individuals working in the same field that wish to build on pre-existing innovation, and enables effective investment in innovation (Kavuşturan 2020; Fraser 2016). Without disclosure, the patent system would be less effective in fostering innovation.

But if AI-generated inventions cannot be patented, and if humans are discouraged from (falsely) naming themselves as the inventors, the main incentive will be to keep AI-generated inventions as trade secrets and to enjoy trade secrecy protections over these inventions. As a result, the objectives of patent law, to make technical solutions public in exchange for monopolistic rights over that invention, will not be fulfilled. This may lead to an inefficient allocation of financial resources, as multiple actors may be working on the same problem, thereby increasing the overall cost of research for humanity, reducing collaboration in the R&D space, and misaligning investments.

### (v) Rejecting AI inventorship will prevent the advancement of AI-based R&D

In addition to the foregoing somewhat technical arguments, there is the general policy goal to bear in mind. If we care about innovation, and we wish to encourage it for the benefit of humanity, there are good reasons to encourage the use of AI in the innovation process. Some economists and technology experts are already arguing that we are living through an era of reduced innovation and growth. It seems that innovations are becoming harder to make and that more money is being spent to produce fewer net inventions (Gold 2021; Collison and Nielsen 2018; Wong 2017). Given the social, economic, and environmental challenges we face, we need all the help we can get. AI has the potential to speed up innovation. AI systems perform in a much quicker and efficient manner. In addition to this, unsupervised machine learning techniques can enable AI systems to detect patterns or a relationship in data that cannot be noticed by human experts (Alpaydın 2020; Ebrahim 2020; McLaughlin 2018). In this way, AI systems not only detect known-unknowns, but also might enable the discovery of unknown-unknowns (European Patent Office n.d.; Wilson and Daugherty 2019; Mammen and Richey 2020; Fraser 2016; McLaughlin 2018). This could be a major boon to innovation.

Some may counterargue here that intellectual property law already encourages the development of inventive AI through the copyright and patent protection of AI itself. In other words, maybe DABUS’s inventions are not patentable but DABUS itself is (as is AlphaFold) and that could be enough to foster innovation. But these protections alone are insufficient for effective development of inventive AI technologies. If inventions generated by AI cannot benefit from patent protection, the intellectual property safeguard for the inventive AI system becomes ineffective. This is because the defining characteristic of inventive AI is the generation of inventions. If the resulting inventions cannot be protected under the applicable patent law, the value of intellectual property protection for such AI diminishes. The actual use of AI in the innovation process is not encouraged and is even discouraged by the refusal to grant a patent to AI-generated inventions. Why should Thaler persist in using DABUS to assist with invention if the resulting inventions cannot be patented? Thus, rejecting AI inventorship might have the perverse effect of diminishing the efforts of people who are working to develop innovative AI systems. Why bother if any inventions generated by such systems can be expropriated by others?

Currently, natural persons are needed for developing and effectively using inventive AI. AI itself is not motivated to innovate through the patent system, but those who develop inventive AI can be motivated and encouraged to develop inventive AI through effective legal regulation (Abbott 2020; Pearlman 2018). In other words, the legal recognition of the increasing role of inventive AI in the invention process does not eliminate the need for direct incentives for people to develop inventions but rather requires an up-to-date interpretation and implementation of the incentive mechanism set by patent law. This is another reason why inventions created by AI should be patentable.

## Limitations and Counter-Arguments

AI’s inventorship status constitutes a foundational layer for the effective prosecution of patent laws, which will encourage the integration of these technologies into R&D processes. Such encouragement is vital, considering the transformative impact these technologies will have on R&D processes, as discussed above, and their potential to address current challenges, such as pandemics and environmental sustainability. However, while this debate is essential for the incorporation of such technologies and the advancement of R&D processes, a focus on the patent protection of AI-generated inventions—specifically, the inventorship status of these technologies—only addresses a limited aspect of integrating AI into today’s world. Recognizing AI systems as inventors and incorporating these technologies into R&D will not resolve all the challenges posed by AI or solve all the problems of the twenty-first century at once. Thus, this reform alone will not be sufficient; and complementary legal policies must accompany it to foster a world where AI technologies are harnessed for humanity’s benefit and the challenges they present are effectively managed.

There are certain general concerns regarding the development of AI technologies, such as the environmental cost of the technology, the privacy breaches it enables, the potential for these technologies to make humans less intelligent or creative, and the unemployment or power shift concerns (Sharma 2024; Farhan 2023; Ivanov 2024; Samore 2013). All these concerns may apply to inventive AI as well. There is not the space to address all these criticisms and concerns in this article. However, we will conclude by addressing some concerns and counter-arguments specifically arising in relation to AI’s inventorship status. Some may argue that recognizing AI systems as inventors could trigger the following challenges: (i) complex patent application processes, (ii) challenges in the correct attribution of patent ownership, (iii) divergences in how AI and humans act in R&D processes, and (iv) monopolization of R&D markets.

### (i) Complex patent application processes

In principle, the right to apply for a patent lies with the inventors or non-inventors who can assign this right through certain legal processes, which include contracts and the rule of law in many jurisdictions. However, it is not clear how the right to apply for a patent will be borne for AI-generated inventions, and this has been discussed in the cases brought for the inventions generated by DABUS. That’s why some may argue that the AI’s inventorship status will complicate the patent application processes, and that keeping the status quo will be better for the efficiency of the system.

However, this argument is flawed. The argument is based on the assumption that if the inventorship status is not open to AI technologies, these technologies will not be used in the R&D processes. It seems more likely that they will be used but that their use will be concealed or hidden in some way. Thus, if the status quo is chosen, issues regarding the use of AI in R&D are more likely to be taken to the courts (or litigation is more likely to be threatened or feared), and the resulting conflicts will be resolved through rules that lack a true recognition of the realities of today’s R&D. This litigation risk will increase the complexity and inefficiency of the existing system.

Formally recognizing AI’s inventorship status will lead to necessary discussions in legislative bodies and will prevent a portion of the conflict that might arise due to uncertainties about the allocation of the right to apply for a patent for such AI-generated inventions.

### (ii) Challenges in the correct attribution of patent ownership

If AI can be recognized as an inventor, there is still the issue of who should own the resulting patent. Remember, we are not arguing here that AIs themselves should be the owners. Humans and human-run enterprises would continue to own patents. But given the complex nature of AI development, and the webs of technology underlying a single AI system, disputes may arise as to which of these human-run organisations should own a patent. This might be used as grounds to maintain the status quo.

But, as already noted in our discussion of AlphaFold, recognizing AI systems as inventors does not create these challenges but rather makes them visible. The complexity of developing AI technologies, along with the various stakeholders involved in their development and deployment, complicates the attribution of ownership to these parties. Database providers, algorithm developers, R&D groups as users, their employers, and the entity controlling the inventive AI technology may possibly share the ownership of patents, depending on their material contribution to the creation of a specific invention (Pearlman 2018; Schuster 2018; Mazzi 2020).

The level of contribution and the value that these actors bring to the invention will be crucial in the discussion of patent ownership. Additionally, the complex relationships among these parties pose legal challenges regarding the contractual provisions in their licensing agreements. Therefore, it is legally significant to consider what kinds of contractual agreements would be valid before a patent office when applying for a patent based on a contractual relationship between the parties.

However, these discussions exceed the scope of this article. For the purposes of this article, it should be highlighted that AI's inventorship is not the source of these challenges but rather a means of recognizing the inherent complexity of these challenges.

### (iii) Divergences in how AI and humans act in R&D processes

Some people may argue that AI is not creative and innovative in the way humans are, and that’s why AI systems cannot be recognized as inventors (Knutson 2020). This is probably true; AI systems do not follow the same mechanisms that the human brain is based on, and the process of AI invention may be very different to that of human invention. Our point is that this does not matter; what matters is the value and equality of the inventions that are created by these systems.

Patent law is the guardian of technical innovation. The economic rationale behind patent law is the market failure called the appropriability dilemma (Cuntz, Fink, and Stamm 2024). This failure refers to the situation where knowledge is out in public, and the marginal cost of getting that information is near to zero due to the nonrival and non-excludable nature of knowledge (Fisher 2007; Cuntz, Fink, and Stamm 2024). This creates an unjust situation for those who have invested time and money in the research and development process, which led to the technical knowledge that serves a technical solution. The inclusion of AI technologies in R&D processes does not eliminate this market failure (Cuntz, Fink, and Stamm 2024). So, since the economic rationale continues to exist, not patenting AI-generated inventions would not seem to be the right policy economically (Cuntz, Fink, and Stamm 2024). For this reason, AI-generated inventions need to be patented as long as the economic rationale persists even though they are not directly the products of the human innovation.

### (iv) Monopolization of R&D markets

It is true that developing innovative AI technologies is neither mainstream nor easy. The cost of training these technologies is high, and only a few companies have the capacity to develop and deploy them (Cuntz, Fink, and Stamm 2024). Recognizing AI systems as inventors might encourage the integration of AI into R&D processes; however, this could also lead to the elimination of small and medium enterprises in this market due to the dominance of these larger corporations. This could result in increased monopolization and consolidation of the power in the hands of a few companies. The proposed legal change may, consequently, benefit these big companies, but not society as a whole.

This argument is valid to a certain degree; however, it needs to be appropriately contextualized. It is true that there are high costs associated with the development of AI technologies, and only a few companies have access to these innovative AI technologies or the capacity to develop them. This will certainly decrease competition in the relevant market. However, this decrease in competition will not be due to the *recognition* of these technologies as inventors, but rather due to the development of these technologies.

AI technologies are developing regardless. Thus, recognizing AI’s inventorship status will not monopolize the market, but rather will clarify and substantiate any monopolization due to the transparency it will facilitate. This will provide the basis for using competition law tools to combat the downsides of this concentration in the relevant markets.

According to the well-established economic principle of economies of scale, where fixed costs are high, the marginal cost of producing goods or services decreases as production increases. Considering the high costs of developing inventive AI technologies and the complexity of the process (Cuntz, Fink, and Stamm 2024), it does not seem economically reasonable for each actor in the AI development market to develop their own inventive AI systems. To benefit from economies of scale, inventive AI technologies should be available to be used by several R&D companies through the operation of licensing agreements. In this way, R&D groups would be allowed to use these technologies to produce more technical solutions. This could be legally mandated under the “essential facilities” doctrine (Mazzi 2020). This is a legal doctrine used in many jurisdictions to require dominant market players to grant access to certain essential facilities to competitors. Recognizing AI technologies as inventors will facilitate the application of the essential facilities doctrine to the technology, ensuring a legal and economic structure that benefits society.

Competition is not an ideal that legal systems strive to achieve at all costs; rather, it is a tool aimed at achieving the public good. However, under certain circumstances, the nature of the market and the costs associated with a particular good or service can make competition economically unreasonable. Sometimes, the public good is established through legal recognition of an economic reality. For example, due to network externalities, the transmission and distribution of electricity is provided by a regulated monopoly. This legal recognition of economic facts allows legal systems to achieve the public good. Thus, the recognition of AI systems’ inventorship status will bring transparency, which may not prevent the monopolization that will occur after the development and deployment of these technologies in R&D processes; however, it will prevent future monopolization in subsequent markets and allow small and medium companies to utilize these technologies in their R&D, benefiting overall invention generation.

## Conclusion

In current patent office evaluations and court verdicts, the consensus is that AI systems are not natural persons and so they cannot be recognized as inventors for the purpose of granting patent rights. We have argued that this approach is mistaken because it is not in compliance with the spirit or purpose of the patent system. Maybe one day the advancement and the intervention of AI will come to a point where patent law will become redundant since innovation will become self-sustaining: the machines will generate more inventions without the need for human-friendly incentives. That is not the world in which we currently live. In today’s world, a transition period (Abbott 2020) is occurring in which human inventors work alongside AI inventors. In this transitional world, patent law is still required but needs to be updated by taking AI-generated inventions seriously, recognizing the potential inventorship status of AI, and updating patentability criteria appropriately in light of recent technological developments.

## Data Availability

There is no data associated with this article.

## Footnotes

1 The patent for the inventions generated by DABUS and naming DABUS as the inventor can be accessed at https://iponline.cipc.co.za/Publications/PublishedJournals/E_Journal_July%202021%20Part%202.pdf (255). For detailed information on the applications see https://artificialinventor.com/patent-applications.

2 Thaler v Comptroller-General of Patents, Designs and Trademarks [2023] UKSC 49

3 *European Patent Office Board of Appeal Decision J 0008/20 (Designation of inventor/DABUS)* [2021]; *Thaler v Vidal*, 21-2347 (Fed Cir, 2022).

4 Thaler v Vidal, Petition 22-919 (2023).
